# Selective inhibitors of phosphoinositide 3-kinase delta: modulators of B-cell function with potential for treating autoimmune inflammatory diseases and B-cell malignancies

**DOI:** 10.3389/fimmu.2012.00256

**Published:** 2012-08-23

**Authors:** Kamal D. Puri, Michael R. Gold

**Affiliations:** ^1^Gilead Sciences, Inc., SeattleWA, USA; ^2^Department of Microbiology and Immunology, I^3^ and CELL Research Groups, Life Sciences Institute, University of British ColumbiaVancouver, BC, Canada

**Keywords:** phosphoinositide 3-kinase, p110delta, autoimmunity, GS-1101, CAL-101, IC87114, non-Hodgkin lymphoma, leukemia

## Abstract

The delta isoform of the p110 catalytic subunit (p110δ) of phosphoinositide 3-kinase is expressed primarily in hematopoietic cells and plays an essential role in B-cell development and function. Studies employing mice lacking a functional p110δ protein, as well as the use of highly-selective chemical inhibitors of p110δ, have revealed that signaling via p110δ-containing PI3K complexes (PI3Kδ) is critical for B-cell survival, migration, and activation, functioning downstream of key receptors on B cells including the B-cell antigen receptor, chemokine receptors, pro-survival receptors such as BAFF-R and the IL-4 receptor, and co-stimulatory receptors such as CD40 and Toll-like receptors (TLRs). Similarly, this PI3K isoform plays a key role in the survival, proliferation, and dissemination of B-cell lymphomas. Herein we summarize studies showing that these processes can be inhibited *in vitro* and *in vivo* by small molecule inhibitors of p110δ enzymatic activity, and that these p110δ inhibitors have shown efficacy in clinical trials for the treatment of several types of B-cell malignancies including chronic lymphocytic leukemia (CLL) and non-Hodgkin lymphoma (NHL). PI3Kδ also plays a critical role in the activation, proliferation, and tissue homing of self-reactive B cells that contribute to autoimmune diseases, in particular innate-like B-cell populations such as marginal zone (MZ) B cells and B-1 cells that have been strongly linked to autoimmunity. We discuss the potential utility of p110δ inhibitors, either alone or in combination with B-cell depletion, for treating autoimmune diseases such as lupus, rheumatoid arthritis, and type 1 diabetes. Because PI3Kδ plays a major role in both B-cell-mediated autoimmune inflammation and B-cell malignancies, PI3Kδ inhibitors may represent a promising therapeutic approach for treating these diseases.

## The role of PI3K signaling in B-cell development and activation

B cells play a critical role in immune system function and dysfunction (e.g., autoimmunity) by producing antibodies and by acting as antigen-presenting cells (APCs) for T cells. Signaling via phosphoinositide 3-kinase (PI3K) controls many essential B cell functions and is therefore a promising target for preventing aberrant B cell activation. The class I PI3K enzymes consist of a regulatory subunit that allows receptors to recruit PI3K to the plasma membrane, and a catalytic subunit, which can then phosphorylate phosphoinositide lipids on the 3' position of their inositol head group. The lipid second messengers generated by PI3K, primarily phosphatidylinositol 3,4,5-trisphosphate (PIP_3_), can recruit cytosolic signaling enzymes that contain pleckstrin homology (PH) domains to the plasma membrane (Lemmon, 2008). The resulting formation of protein complexes facilitates the activation of these signaling enzymes and brings them in close proximity to their substrates. The PI3K “effectors” that are recruited to the plasma membrane and activated in this manner include signaling enzymes that control cell survival, activation growth, proliferation, and differentiation, as well as cell motility, cell adhesion, and specialized processes such as phagocytosis (Okkenhaug and Vanhaesebroeck, 2003; Fruman, 2004). The structure, enzymatic activity, and functions of the class I PI3Ks have been described in detail in many excellent reviews (Okkenhaug and Fruman, 2010; Vanhaesebroeck et al., 2010; So and Fruman, 2012).

PI3K signaling is important for B-cell development (Fruman et al., 1999; Suzuki et al., 1999; Clayton et al., 2002; Okkenhaug et al., 2002) due its role in mediating the survival and differentiation signals that are initiated by the pre-B-cell receptor (Ramadani et al., 2010). In mature B cells, PI3K transduces signals from a wide variety of receptors that control nearly all aspects of B-cell function. The survival of naïve B cells depends on constitutive low-level antigen-independent activation of PI3K by the B-cell receptor (BCR) (Srinivasan et al., 2009) and by the receptor for the cytokine B-cell activating factor (BAFF) (Henley et al., 2008). These survival signals may be mediated, at least in part, by the Akt protein kinase, a major downstream target of PI3K signaling. Akt activates multiple pro-survival pathways while inhibiting pro-apoptotic pathways.

**Figure 1:**
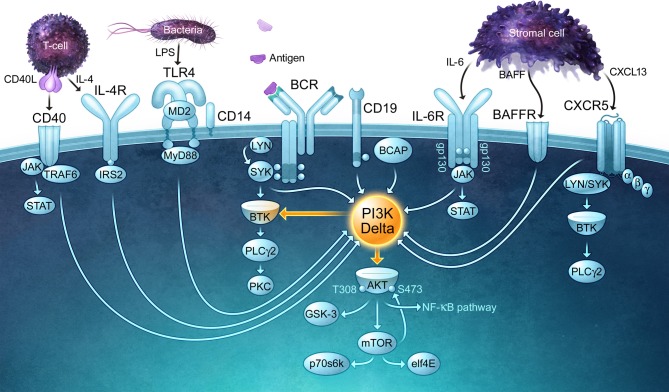
**PI3Kδ is a central signaling enzyme that mediates the effects of multiple receptors on B cells.** PI3Kδ signaling is important for B-cell survival, migration, and activation, functioning downstream of the B-cell antigen receptor (BCR) and its co-receptor CD19, chemokine receptors (CXCR5), and activating/co-stimulatory receptors such as CD40 and Toll-like receptors (TLRs). Cytokines derived from lymphoid stromal cells (BAFF, IL-6) and T cells (IL-4) that are essential for the expansion and survival of B cells also require PI3Kδ for their actions and bind receptors that activate PI3Kδ. The receptors depicted in this figure use a variety of kinases and adaptor proteins to recruit PI3Kδ to the plasma membrane, where it can produce the lipid second messenger PIP_3_. By binding to PH domain-containing proteins, PIP_3_ promotes the activation of multiple signaling enzymes including PLCγ 2 and Akt, both of which control key signaling networks. Note that the connecting arrows may represent multiple intermediate signaling reactions.

The detection of foreign antigens by circulating B cells depends on the ability of B cells to traffic into lymphoid organs and migrate into the lymphoid follicles, where antigens are captured and retained via multiple mechanisms (Batista and Harwood, 2009). The *in vivo* trafficking of B cells is directed by chemokines such as CXCL13 and CCL21 as well as the lipid chemoattractant sphingosine 1-phosphate (S1P) (Stein and Nombela-Arrieta, 2005). The G protein-coupled receptors that bind chemoattractants activate PI3K, and this is critical for B cells to migrate towards these stimuli. Once B cells encounter a foreign antigen, which in the case of an infection will occur in the presence of microbially-derived ligands for Toll-like receptors (TLRs), PI3K signaling is essential for B-cell activation and proliferation as well as the subsequent differentiation of B cells into antibody-producing cells and the survival of memory B cells. As described in detail elsewhere (Fruman, 2004), PI3K plays a central role in the activation of many BCR signaling pathways. In particular, PIP_3_-dependent activation of Bruton's tyrosine kinase (Btk) is crucial for the activation of phospholipase C-γ, an enzyme that splits phosphatidylinositol 4,5-bisphosphate (PIP_2_) into inositol 1,4,5-trisphosphate (IP_3_) and diacylglycerol, second messengers that lead to increases in intracellular Ca^2+^, activation of multiple protein kinase C isoforms, activation of the NF-κB and NF-AT transcription factors, and activation of the Ras and Rap1 GTPases, the latter of which is a master regulator of cytoskeletal reorganization and integrin-mediated adhesion. B-cell activation also requires critical input from co-stimulatory receptors such as CD40 and the IL-4 receptor, which transduce signals from helper T cells, as well as TLRs, which are now thought of as a third signal for B-cell activation. CD40, the IL-4 receptor, and the main TLRs expressed by B cells, TLR4 and TLR9, all signal via PI3K (Figure 1). Not only does PI3K signaling act downstream of multiple receptors that drive different steps in the B-cell activation process (Donahue and Fruman, 2004), it is also important for B cells to bind to T cells so that they can act as APCs and elicit T cell help in the form of CD40 ligand and IL-4 (Al-Alwan et al., 2007).

## Isoform-specific functions: a critical role for PI3Kδ in B cells

There are four isoforms of class I PI3K catalytic subunits: p110α, p110β, p110γ, and p110δ. Although these enzymes appear to have identical substrate specificity, they clearly have distinct functions *in vivo*, as revealed by the generation of mouse strains in which the genes encoding these different catalytic subunits have either been disrupted or replaced by a catalytically inactive version (Rommel et al., 2007; Vanhaesebroeck et al., 2010; So and Fruman, 2012). This could be explained in part by differential expression or abundance of the various isoforms in specific tissues. In particular, p110γ and p110δ are expressed primarily in hematopoietic cells and have important roles in the functions of both innate and adaptive immune cells (Rommel et al., 2007). It is more difficult to explain how different PI3K isoforms can play distinct roles within the same cell. Proposed models usually invoke preferential recruitment of specific PI3K isoforms by different receptors or to different membrane domains, which results in the formation of distinct signaling complexes. This would presumably depend on unique structural features of the different catalytic subunits mediating specific interactions.

An intriguing finding is that p110α is important for the tonic antigen-independent survival signals generated by the pre-BCR and BCR whereas p110δ (but not p110α) is required for antigen-dependent B-cell activation driven by the BCR (Ramadani et al., 2010). These distinct roles of p110α and p110δ in homeostatic versus antigen-induced BCR signaling could reflect different mechanisms by which PI3K complexes are recruited to the plasma membrane in resting versus activated B cells. The TC21 GTPase can directly bridge PI3K catalytic subunits to the non-phosphorylated BCR immunoreceptor tyrosine-based activation motifs (ITAMs) in resting B cells, thereby promoting homeostatic PI3K signaling (Delgado et al., 2009). In contrast, antigen-induced BCR clustering, and the resulting tyrosine kinase activation and ITAM phosphorylation, could abrogate the binding of TC21 to the BCR and instead promote SH2-mediated binding of PI3K to BCAP and CD19, scaffolding proteins that are tyrosine phosphorylated after BCR engagement. This model would require that TC21 preferentially recruit p110α-containing PI3K complexes whereas BCAP and CD19 preferentially recruit p110δ-containing PI3K complexes. The finding that TC21 recruits p110δ to the BCR in resting B cells (Delgado et al., 2009) does not support this idea. However, this group did not assess whether p110α was also recruited to the BCR by TC21. Thus, the basis for the differential role of p110α and p110δ in homeostatic versus antigen-initiated BCR signaling remains to be determined.

Although B cells express all four isoforms of the class I PI3K p110 catalytic subunit (Bilancio et al., 2006), the p110δ isoform has an essential and non-redundant role in many aspects of B cell development and activation. In mice lacking a functional p110δ protein, fewer mature circulating B cells (i.e., follicular or B-2-B cells) are generated and the B cells that do develop exhibit impaired chemokine-induced migration, BCR signaling, and BCR-induced proliferation, which correlates with reduced differentiation into antibody-producing cells and reduced serum Ig levels both before and after immunization (Clayton et al., 2002; Jou et al., 2002; Okkenhaug et al., 2002; Reif et al., 2004). This is in contrast to knockout mice that lack the leukocyte-specific p110γ catalytic subunit, which is often linked to signaling by G protein-coupled receptors. Even though p110γ is normally expressed in B cells, mice lacking p110γ have no apparent defects in B-cell development or function (Sasaki et al., 2000). PI3Kδ also appears to have a unique role in B-cell development and function in humans. Several genetic polymorphisms in the p110δ gene have been identified and a patient with a primary B-cell immunodeficiency was found to have a missense mutation that resulted in an aspartic acid to lysine replacement within the catalytic domain of p110δ (Jou et al., 2006).

Highly selective small molecule inhibitors of p110δ such as IC87114 (Sadhu et al., 2003a,b) and GS-1101, which is also known as CAL-101 (Lannutti et al., 2011), allow inhibition of this PI3K isoform both *in vivo* and in cells derived from normal animals. The inhibitory effects of IC87114 phenocopy the effects of disrupting the p110δ gene or replacing it with a mutant version that encodes a catalytically inactive form of p110δ (Okkenhaug et al., 2002; Bilancio et al., 2006), supporting the idea that this drug is highly selective for p110δ and can therefore be used as a probe for p110δ function. The use of IC87114 has provided significant insights into the role of p110δ in mature B cells. *In vitro*, treating murine splenic B cells with IC87114 significantly reduces IL-4-dependent B-cell survival, CXCL13-induced B-cell migration, the ability of B cells to present antigens to T cells, TLR-induced cytokine production, and both BCR- and TLR-mediated B-cell activation, proliferation, differentiation, and antibody secretion (Bilancio et al., 2006; Dil and Marshall, 2009; Durand et al., 2009). IC87114 is also highly active *in vivo* and we have shown that it can reduce antigen-specific antibody responses in rodents (Durand et al., 2009).

## The role of PI3Kδ in the development, localization, and function of innate-like B cells

The majority of B cells are conventional circulating B-2 cells. However the marginal zone (MZ) B cells and B-1 subsets also play important roles in host defense (Pillai et al., 2005; Baumgarth, 2011). B-1 cells are found in the peritoneal cavity and the spleen, whereas MZ B cells reside near the marginal sinus of the spleen where they are positioned to rapidly detect blood-borne microbial infections. The Ig repertoires of B-1 and MZ B cells are skewed toward the recognition of both microbial antigens and self-antigens. So-called natural antibodies that are produced by these B-cell subsets in the absence of any antigen stimulation (as well as in germ-free mice) recognize cross-reactive epitopes on Gram-positive bacteria, viruses, apoptotic cells, and oxidized low-density lipoproteins (Ochsenbein et al., 1999; Zhang and Carroll, 2007; Binder et al., 2008). This provides protection against infection and also prevents inflammation by clearing oxidized lipids, oxidized proteins, and apoptotic cells (Binder et al., 2008; Chou et al., 2008; Baumgarth, 2011). In addition, exposure to TLR ligands leads to a rapid increase in antibody production by B-1 and MZ B cells. Hence, these B-cell subsets have been termed “innate-like” B cells because of their constitutive production of protective antibodies, their ability to mount rapid responses to infection, and their localization to specific sites where they act as sentinels of infection.

A striking observation is that mice in which the gene encoding the p110δ subunit has been disrupted or replaced with a catalytically inactive version have very few B-1 or MZ B cells (Clayton et al., 2002; Okkenhaug et al., 2002). The reasons why the development of these B-cell subsets is strongly dependent on p110δ are not fully understood although PI3K-dependent Akt activation appears to be more important for the development of B-1 and MZ B cells than conventional B cells. Irradiated mice reconstituted with hematopoietic progenitors that lack both the Akt1 and Akt2 isoforms develop normal numbers of circulating B-2 cells but lack MZ B cells and have greatly reduced numbers of B-1 cells (Calamito et al., 2010). The self-reactive nature of B-1 and MZ B cells has led to the idea that B-cell progenitors are directed into these lineages by weak BCR signals initiated by the binding of self-antigens, which could induce PI3K signaling. Where and when this self-antigen encounter occurs is not clear. B-1 cells are thought to arise from a unique population of committed B-1 progenitor cells that retain self-renewal capacity (Montecino-Rodriguez et al., 2006). MZ B cells arise from transitional cells in the spleen that can give rise to either conventional “follicular” B-2 cells or to MZ cells, a cell fate decision in which Notch signaling promotes the MZ B cell fate (Pillai and Cariappa, 2009). The role of PI3Kδ signaling in these cell fate decisions is not known, although it has been proposed that FOXO transcription factors, which are inhibited by PI3K-Akt signaling, may oppose the effects of Notch signaling (Chen et al., 2010).

Because B-1 and MZ B cells are nearly absent in mice lacking a functional p110δ protein, we have used the p110δ-selective inhibitor IC87114 to assess the role of this PI3K isoform in B-1 and MZ B cells that had developed in normal wild-type mice. These studies revealed a number of critical roles for p110δ activity in innate-like B cells (Durand et al., 2009). The localization of innate-like B cells is critical for their ability to mount rapid T cell-independent antibody responses against pathogens. For MZ B cells, their location near the marginal sinus of the spleen positions them to rapidly detect blood-borne pathogens. The retention of MZ B cells in this region surrounding the B-cell follicles is maintained by integrin-mediated adhesion and by opposing chemoattractant gradients established by the chemokine CXCL13, which is produced by follicular dendritic cells within the B-cell follicle and the lipid chemoattractant S1P, which is present at high concentrations in the blood (Cinamon et al., 2004). In response to TLR ligands as an indicator of infection or tissue damage, MZ B cells can move into the B-cell follicles and may eventually exit the spleen and migrate to other tissues in response to CXCL13 (Marino et al., 2008; Rubtsov et al., 2008). Similarly, the mobilization of peritoneal B-1 cells to mucosal sites of infection is stimulated by TLR ligands and depends on a robust migratory response to the chemokine CXCL13 (Ha et al., 2006). Our *in vitro* studies showed that p110δ activity is important for B-1 cells to migrate toward CXCL13 and for MZ B cells to exhibit chemotactic responses to both CXCL13 and S1P (Durand et al., 2009). In addition, CXCL13-stimulated adhesion of MZ B cells to the integrin ligand ICAM-1 was reduced to below basal level when these cells were treated with IC87114 (Durand et al., 2009). Consistent with the finding that *in vitro* treatment of MZ B cells with IC87114 inhibited both chemoattractant-induced migration and adhesion, oral administration of IC87114 to mice dramatically reduced the number of MZ B cells surrounding the B-cell follicles of the spleen, similar to what is seen in mice lacking a functional p110δ protein (Durand et al., 2009).

What are the molecular mechanisms by which PI3Kδ signaling promotes the cell motility and cell adhesion that control the localization of MZ B cells? CXCL13- and S1P-induced B-cell migration and adhesion are strongly dependent on activation of the Rap1 GTPase (Durand et al., 2006), a master regulator of cytoskeletal organization, cell polarity, cell motility, and integrin activation (Bos, 2005). Our finding that p110δ activity is required for CXCL13 and S1P to activate Rap1 in B cells (Durand et al., 2009), combined with the observation that Rap1b-deficient mice have greatly reduced numbers of MZ B cells (Chen et al., 2008), suggests that p110δ-dependent activation of Rap1b is essential for the *in vivo* localization of MZ B cells.

PI3Kδ signaling also plays an essential role in the activation of innate-like B cells. Treating murine splenic MZ B cells or B-1 cells with either IC87114 or the pan-PI3K inhibitor LY294002 cause nearly complete inhibition of Akt phosphorylation that is stimulated by CXCL13, by the TLR9 ligand CpG DNA, or anti-Ig antibodies that cluster the BCR. IC87114 also inhibits S1P-induced Akt phosphorylation in MZ B cells (neither B-1 nor B-2 cells respond well to S1P). These findings suggest that p110δ is the main PI3K isoform linking the BCR, TLR9, and the receptors for CXCL13 and S1P to activation of the Akt pro-survival kinase in innate-like B cells. This is somewhat surprising with regard to chemoattractant signaling since G protein-coupled receptors such as those that bind CXCL13 and S1P are often coupled to PI3Kγ. PI3Kγ is expressed in B cells (Bilancio et al., 2006), although this analysis has not been carried out on isolated B-1 or MZ B cell populations. However, mice lacking p110γ have no apparent defects in B-cell development or function (Sasaki et al., 2000), suggesting that p110δ substitutes for p110γ in linking G protein-coupled receptors to the PI3K/Akt pathway in B cells. Befitting their role as sentinels that provide early detection and protection from infection, innate-like B cells are most frequently activated in a polyclonal manner by TLR ligands. The ability of the TLR ligands lipopolysaccharide (LPS) and CpG DNA to induce the proliferation of B-1 and MZ B cells and to increase antibody secretion by purified splenic MZ cells or peritoneal B-1 cells is substantially reduced in the presence of the p110δ inhibitor IC87114 (Durand et al., 2009). Recent work has suggested the TLRs may activate p110γ in myeloid cells (Schmid et al., 2011) but its contribution to TLR-induced activation of innate-like B cells has not been assessed. Nevertheless, it is clear that p110δ activity is critical for the activation of innate-like B cell populations by chemokines, antigens, and TLR ligands.

Taken together, these data suggest that p110δ inhibitors such as IC87114 may be useful for inhibiting the *in vivo* trafficking, localization, and activation of MZ B cells and B-1 cells, innate-like B-cell populations that have been strongly implicated in a variety of autoimmune diseases.

## PI3Kδ as a target for treating B cell-mediated autoimmune diseases

Autoimmune inflammatory diseases such as rheumatoid arthritis, systemic lupus erythematosus (SLE), and type 1 diabetes affect millions of people worldwide. The aging population, especially in the developed world, as well as complex environmental factors, has contributed to a significant increase in the number of patients requiring treatment. Although there have been dramatic advances in the development of biological treatments in the form of antibodies and recombinant proteins, not all patients experience effective disease management. Hence there continues to be a need for new treatment approaches that provide effective long-term protection from disease progression with minimal side effects, simple delivery, and reasonable cost.

### The role of B cells in chronic autoimmune inflammatory diseases

B cells play a major role in a number of autoimmune diseases via their production of autoantibodies, their ability to act as APCs that present self-antigens to autoreactive T cells, and their production of pro-inflammatory cytokines (Murakami and Honjo, 1997; Fields and Erikson, 2003; Martin and Chan, 2006; Shlomchik, 2008; Yanaba et al., 2008). The role of B cells in the pathogenesis of rheumatoid arthritis, type 1 diabetes, multiple sclerosis, SLE, and several other autoimmune inflammatory diseases has been described in detail elsewhere (Yanaba et al., 2008).

Aberrant B-cell activation and polyclonal antibody production can occur when there are defects in negative regulators of BCR signaling such as the Shp1 tyrosine phosphatase. In mice, the loss of Shp1 only in B cells is sufficient to cause an SLE-like disease (Pao et al., 2007). Viral and bacterial infections may also trigger autoimmunity by causing polyclonal activation of B cells via TLRs. In contrast to polyclonal B-cell activation, the loss of B-cell tolerance due either to intrinsic defects or to aberrant T-cell activation can result in the production of characteristic autoantibodies, for example the “rheumatoid factor” anti-IgG antibodies that are associated with rheumatoid arthritis. Autoantibodies can greatly amplify inflammatory responses and thereby play an important role in promoting tissue damage. Immune complexes containing IgM or IgG autoantibodies can initiate complement-mediated inflammation and can activate inflammatory cells by binding to Fc receptors. The formation of large immune complexes can also cause blockages that lead to glomerulonephritis or thrombosis (Lipsky, 2001). Finally, autoantibodies that bind to host cells may promote antibody-dependent cellular cytotoxicity carried out by NK cells or macrophages.

More recent data have shown that B cells can also initiate and amplify autoimmune disease via their ability to present antigens to T cells. This has been demonstrated convincingly in the non-obese diabetic (NOD) mouse model of spontaneous autoimmune type 1 diabetes. NOD mice that lack B cells (NOD μMT mice) do not develop diabetes (Serreze et al., 1996). However, NOD mice in which B cells are present but cannot secrete antibodies develop diabetes to the same extent as wild-type NOD mice (Wong et al., 2004). This suggests that the major role for B cells in autoimmune diabetes is as APCs. Consistent with this conclusion, B cells are required for the proliferation of diabetogenic T cells in NOD mice (Katz et al., 1993; Serreze et al., 1996; Falcone et al., 1998; Noorchashm et al., 1999a; Tian et al., 2006; Marino and Grey, 2008) and for the activation and survival of CD8^+^ T cells that infiltrate the pancreas and attack the insulin-producing beta cells (Brodie et al., 2008). The hyperactivated B cells present in autoimmune animals and patients are highly effective APCs because they express elevated levels of MHC proteins and the T cell co-stimulatory molecules CD80 and CD86 on their surface.

Cytokine production by B cells may also contribute to the progressive amplification of autoimmune processes. Activated B cells can produce pro-inflammatory cytokines such as IL-6 (Shlomchik, 2008; Yanaba et al., 2008). Moreover, in chronic inflammatory autoimmune diseases such as rheumatoid arthritis, lymphotoxin-β produced by B cells promotes the formation of ectopic GCs within the inflamed tissue (Takemura et al., 2001; Bugatti et al., 2007). Activated B cells that enter these germinal centers undergo clonal expansion, somatic hypermutation, and affinity maturation, leading to increased production of higher affinity self-reactive antibodies (Kim and Berek, 2000).

### The role of innate-like B cells in autoimmunity

In a number of mouse models of autoimmune inflammatory diseases such as rheumatoid arthritis and SLE, disease progression is strongly associated with an expansion of B-1 and MZ B cell populations and a corresponding increase in the production of self-reactive antibodies that may contribute to the autoimmune pathology (Batten et al., 2000; Duan and Morel, 2006; Ishida et al., 2006; Bugatti et al., 2007; Pao et al., 2007; Marino et al., 2008).

The role of innate-like B cells in autoimmunity has been characterized extensively in mouse models of autoimmune diabetes that closely resemble autoimmune type 1 diabetes in humans. In type 1 diabetes, T cells specific for pancreatic beta cells become activated and infiltrate the islets of Langerhans. CD8^+^ cytotoxic T cells directly kill beta cells while pathogenic CD4^+^ T_H_1 cells recruit macrophages that produce inflammatory mediators such as IL-1β, TNF-α, and nitric oxide, which combine to cause beta cell dysfunction and death. The net result is progressive destruction of the beta cells, leading to insulin deficiency and hyperglycemia. At least in the NOD mouse model of spontaneous autoimmune diabetes, innate-like B cells appear to play an important role in disease progression. NOD mice have increased numbers of MZ B cells (Marino et al., 2008), a phenotype that maps to the Idd9/11 diabetes susceptibility locus (Rolf et al., 2005). Prior to the onset of diabetes in NOD mice, activated MZ B cells accumulate in the pancreatic lymph node (Marino et al., 2008), the main site of activation of diabetogenic T cells. MZ B cells are efficient APCs (Attanavanich and Kearney, 2004), express T cell co-stimulatory molecules when activated (Oliver et al., 1999; Wither et al., 2000; Marino et al., 2008), and can effectively present beta cell antigens to self-reactive T cells from NOD mice and induce their proliferation (Marino et al., 2008). Consistent with a role for MZ B cells in the development of autoimmune diabetes, the somewhat selective depletion of MZ B cells with antibodies to CD21/CD35 reduces the disease incidence in a mouse model (Noorchashm et al., 1999b). B-1 cells may also promote the autoimmune destruction of islet cells. B-1 cells are found primarily in the peritoneal cavity but have been shown to traffic to the pancreas prior to the onset of type 1 diabetes in a mouse model of the disease (Ryan et al., 2010). In the pancreas, B-1 cells promote the infiltration of diabetogenic T cells by inducing the pancreatic vasculature to express VCAM-1 (Ryan et al., 2010), an integrin ligand that supports T cell extravasation. Thus the combined actions of MZ B cells and B-1 cells may be a key factor in the activation of diabetogenic T cells and their ability to infiltrate the pancreas, where they orchestrate the destruction of insulin-producing islet cells.

The role of innate-like B cells in autoimmune diseases in humans has yet to be fully explored because the equivalent cell populations have been more difficult to identify. A CD27^+^ human B-cell subset has been likened to murine MZ B cells in that these cells can generate rapid T-independent antibody responses against the carbohydrate capsules of pathogenic Gram-positive bacteria. However, unlike murine MZ B cells that are localized mainly to the spleen and express an “innate” germ-line encoded Ig repertoire, CD27^+^ human B cells circulate and their Ig repertoire appears to have undergone antigen-independent somatic hypermutation that may be similar to the way that the B cell Ig repertoire is generated in sheep and other species (Weill et al., 2009). These cells may therefore represent an innate-like B-cell population that does not have an equivalent in the mouse. It is not known whether their Ig repertoire is skewed towards self-reactivity or if they contribute to autoimmunity. In contrast, two distinct subpopulations of human B-1 cells can be distinguished by surface markers and transcriptome profile and a recent report has shown that the CD11b^+^ human B-1 subset can activate T cells and is dramatically increased in SLE patients (Griffin and Rothstein, 2011). The identification of these subsets will undoubtedly lead to further studies of their functions and their potential roles in autoimmunity.

### Targeting B cells to treat autoimmunity

The multiple mechanisms by which B cells contribute to chronic inflammatory autoimmune diseases suggested that B cell depletion could be an effective approach for treating these diseases. Indeed, rituximab, a B cell-depleting anti-CD20 monoclonal antibody that was first developed for the treatment of B-cell malignancies, is now a standard therapy for rheumatoid arthritis (Edwards and Cambridge, 2006). B-cell depletion with monoclonal antibodies against B cell-specific membrane proteins such as CD20 and CD22 is also being considered as a treatment for a variety of other B cell-mediated autoimmune diseases (Kazkaz and Isenberg, 2004; Fiorina et al., 2008; Chan and Carter, 2010). The neutralization of BAFF, a cytokine that promotes the survival of B-2 and MZ B cells may also be a useful approach for depleting autoreactive B cells. Patients with rheumatoid arthritis (Baker, 2004) and SLE (Lebien and Tedder, 2008) exhibit elevated serum levels of BAFF and mice overexpressing BAFF develop SLE-like syndrome with expanded B-cell populations, elevated levels of anti-DNA antibodies, and immune complex deposition in the kidney (Mackay et al., 1999; Khare et al., 2000). In NOD mice, *in vivo* neutralization of BAFF reduces the level of autoantibodies against insulin, decreases the severity of islet inflammation, and reduces the incidence of spontaneous type 1 diabetes (Zekavat et al., 2008). Although global B-cell depletion holds promise as a treatment for autoimmunity, the strong association between autoimmunity and the expansion of innate-like B cells with inherent self-reactivity suggests that inhibiting the activation and function of the human equivalents of MZ B cells and B-1 cells may be an important consideration for effectively treating B cell-mediated autoimmune diseases. In mouse models of autoimmune disease, there is evidence suggesting that selectively targeting innate-like B cell populations can reduce disease incidence and progression. For example, in (NZB × NZW)F_1_ mice, which spontaneously develop autoimmune disease that is very similar to SLE in humans, B-1 cell depletion delays disease onset and reduces disease severity (Murakami et al., 1995), as does depletion of B-1 and MZ B cells using B-cell super antigens (Viau and Zouali, 2005). Similarly, as mentioned above, the somewhat-selective depletion of MZ B cells reduces disease incidence in a mouse model of type 1 diabetes (Noorchashm et al., 1999b). Thus, targeting MZ and B-1 B cells could be a useful approach for treating B cell-mediated autoimmune inflammatory diseases.

### Targeting PI3Kδ to treat autoimmunity

Although a number of different approaches have been used to treat autoimmune diseases, there are currently no curative therapies and it appears that a combination of approaches may be needed to provide effective and long-term protection from disease progression. In the context of treating type 1 diabetes, Luo et al. (2010) have provided a critical analysis of past and current strategies for treating autoimmunity by targeting immune cells. In particular, agents that deplete B cells (e.g., the anti-CD20 monoclonal antibody rituximab) are now being investigated as therapies for B cell-mediated autoimmune diseases. Mouse models of disease first showed the potential of this approach. The incidence of spontaneous autoimmune diabetes in NOD mice can be reduced by depleting B cells with anti-IgM-specific antibodies (Noorchashm et al., 1997), antibodies to CD20 (Hu et al., 2007; Xiu et al., 2008), anti-CD22 antibodies conjugated to a cytotoxic agent (Fiorina et al., 2008), or a fusion protein that sequesters the B-cell survival cytokine BAFF (Marino and Grey, 2008; Zekavat et al., 2008). Similar results have been obtained in other mouse models of autoimmunity (Yu et al., 2008). B-cell depletion with rituximab has now been tested in patients with a variety of B cell-mediated autoimmune diseases, with some success (Kazkaz and Isenberg, 2004; Martin and Chan, 2006; Sanz et al., 2007; Pescovitz et al., 2009; Hegedus et al., 2011). In a phase 2 clinical trial, rituximab treatment partially preserved beta cell function in patients with type 1 diabetes (Pescovitz et al., 2009). This incomplete efficacy could be due the variable penetration of monoclonal antibodies into tissues, resulting in poor depletion on innate-like B cell subsets (Gong et al., 2005; Yu et al., 2008). Moreover, the use of antibodies for treating chronic autoimmune diseases has been associated with infusion site reactions and other adverse reactions caused by immune complex formation (Vogel, 2010; El Fassi et al., 2011). A more serious issue associated with B-cell depletion as a long-term treatment approach for treating autoimmune diseases is chronic immunosuppression (Luo et al., 2010), as well as the potential for eliminating beneficial B cells such as the recently identified “Bregs” that produce IL-10 and limit autoimmune reactions (Mizoguchi and Bhan, 2006; Matsushita et al., 2008). Indeed, in two mouse models of experimental autoimmune encephalomyelitis, anti-CD20-induced B-cell depletion increased the severity of the disease (Weber et al., 2010). The caveats associated with antibody-mediated B-cell depletion strategies suggest that small molecule inhibitors of B-cell function that are orally available, have good tissue penetration, and whose effects are reversible over a short period of time (in contrast to causing long-term immunosuppression) may allow for “tunable” moderate levels of B cell inhibition that can preserve beneficial B-cell functions while having limited side effects over the prolonged treatment course for chronic autoimmune diseases. Such B-cell inhibitors could be useful for managing disease, but may also be useful as an adjunct to rituximab, perhaps by reducing the frequency at which B-cell depletion would need to be done.

Based on the findings described earlier, there are a number of reasons why PI3Kδ is a very promising target for the treatment of autoimmune inflammatory diseases, particularly those in which B cells play a major role. By blocking B-cell migration, adhesion, survival, activation, and proliferation, inhibition of p110δ activity would impair the ability of B cells to act as APCs that activate autoreactive T cells, prevent their activation, and reduces their secretion of autoantibodies and pro-inflammatory cytokines. Moreover, innate-like B cells, which have been strongly linked to autoimmunity, appear to be the most sensitive to loss of PI3Kδ activity as MZ and B-1 cells are nearly absent in mice lacking a functional p110δ gene. We have directly shown that PI3Kδ inhibitors reduce the trafficking and activation of MZ and B-1 cells *in vitro*, as well as the localization of MZ B cells in mice (Durand et al., 2009). Importantly, the restricted expression of p110δ mainly in hematopoietic cells, the observation that B cells are the cell type most impaired in mice lacking a functional version of p110δ, and phase I clinical trial data showing that the PI3Kδ inhibitor GS-1101 is safe and well-tolerated by patients (Kahl et al., 2010) indicate that these compounds can be used to modulate B-cell function *in vivo* without deleterious effects on other cell types or significant side effects. As a proof-of-principle that PI3Kδ inhibitors can inhibit B cell-mediated autoimmune reactions *in vivo*, we showed that administering IC87114 to rats reduces autoantibody production in a model of collagen-induced arthritis (Durand et al., 2009).

PI3Kδ inhibitors such as IC87114 have now been shown to reduce the incidence and severity of autoimmune arthritis (Randis et al., 2008), asthma (Lee et al., 2006; Park et al., 2010), experimental autoimmune encephalomyelitis (Haylock-Jacobs et al., 2011), and SLE (Maxwell et al., 2012) in mouse models. Our current work suggests that oral administration of IC87114 opposes the progression of autoimmune diabetes in NOD mice. The ability of PI3Kδ inhibitors to reduce the severity of these inflammatory diseases may be due not only to their actions on innate-like B cells, but also to inhibitory effects on other immune cells that contribute to autoimmune disease (Fung-Leung, 2011). IC87114 inhibits the trafficking of neutrophils into inflamed tissues (Puri et al., 2004) and prevents the activation of mast cells (Ali et al., 2004, 2008). PI3Kδ also plays an important role in TCR-induced T-cell activation (Okkenhaug et al., 2002, 2006) and IC87114 treatment inhibits TCR-induced cytokine production by both naïve and memory human T cells *in vitro* (Soond et al., 2010). In particular, p110δ signaling is important for IL-17 production by both mouse and human T cells (Park et al., 2010; Soond et al., 2010; Haylock-Jacobs et al., 2011). IL-17-producing T_H_17 cells have been implicated in a number of autoimmune diseases (Jain et al., 2008; Emamaullee et al., 2009; Crome et al., 2010). Finally, PI3Kδ mediates the TCR-induced downregulation of CD62L (Sinclair et al., 2008), which is required for the trafficking of activated T cells into tissues. The broad spectrum of action against cells that contribute to autoimmune processes, combined with the limited expression of p110δ in non-hematopoietic tissues, suggests that PI3Kδ inhibitors could have an excellent therapeutic index for the treatment of autoimmune diseases.

Providing a further rationale for the use of PI3Kδ inhibitors to treat autoimmune disease is the recent finding that enhanced PI3Kδ activity is associated with autoimmunity in humans (Suarez-Fueyo et al., 2011). PI3Kδ activity is significantly increased compared to normal individuals in T cells from ~70% of SLE patients, and this difference is greatest in patients with active disease. Consistent with a role for PI3K in promoting cell survival, SLE patients exhibit an expanded population of CD4+ memory T cells, as well as defective activation-induced cell death (AICD) of T cells, a defect that can be corrected *in vitro* by pharmacological inhibition of p110δ with IC87114. AICD normally limits the expansion of activated T cells and terminates immune responses. Defects in AICD, for example in mice or humans with loss-of-function mutations in Fas or FasL, are strongly correlated with autoimmunity. This suggests that excessive activation of PI3Kδ could contribute to the T cell dysregulation associated with SLE. In this case, PI3Kδ inhibitors may be useful for treating SLE, for which there is currently no effective cure or treatment. The basis for the excessive PI3Kδ activity in SLE patients is not known nor is it known whether aberrant PI3Kδ activity is associated with other autoimmune diseases.

For treating autoimmune diseases, the therapeutic effects of inhibiting PI3Kδ could potentially be enhanced by simultaneously inhibiting PI3Kγ (Rommel et al., 2007). In addition to participating in the activation of T cells through the TCR, PI3Kγ signaling is essential for neutrophils and macrophages to invade tissues and produce inflammatory mediators (Hirsch et al., 2000; Sasaki et al., 2000; Alcazar et al., 2007). Like p110δ, p110γ is expressed mainly in hematopoietic cells, such that selective inhibition of PI3K isoforms containing these catalytic subunits would have limited toxicity on other cell types. Treating mice with the PI3Kγ-selective inhibitor AS605240 can reduce disease incidence and severity in mouse models of rheumatoid arthritis, SLE, and type 1 diabetes (Barber et al., 2005; Camps et al., 2005; Azzi et al., 2012), implicating PI3Kγ in these disease processes and suggesting that PI3Kγ, like PI3Kδ, is a good therapeutic target for treating autoimmune diseases. Abdi and colleagues (Azzi et al., 2012) provided several mechanistic insights into how inhibition of PI3Kγ may prevent the progression of autoimmune diabetes in NOD mice. Both *in vitro* and *in vivo* studies showed that AS605240 inhibited the expansion and effector functions (e.g., cytokine production) of autoreactive CD4+ T cells while expanding the number of regulatory T cells (Tregs), consistent with previous findings that PI3K inhibition causes T cells to become Tregs instead of T-effector cells (Haxhinasto et al., 2008; Sauer et al., 2008) This shift in the T_eff_/Treg balance toward a more immunoregulatory or immunosuppressive state correlated with a delayed onset of diabetes in NOD mice. Importantly, similar to our preliminary findings with the PI3Kδ inhibitor IC87114, treating NOD mice with the PI3Kγ inhibitor when they first exhibited elevated blood glucose levels provided sustained protection from progression to overt diabetes in a number of animals (Azzi et al., 2012). These findings suggest that combined inhibition of PI3Kγ and PI3Kδ could have even greater efficacy in preventing diabetes progression. Recently described dual inhibitors of PI3Kγ and PI3Kδ (Williams et al., 2010) could therefore hold substantial promise as a therapeutic approach for preventing disease progression in patients with early-stage type 1 diabetes and perhaps other chronic autoimmune inflammatory diseases. However dual inhibition of PI3Kγ and PI3Kδ may need to be carefully titrated, as loss of both PI3Kγ and PI3Kδ activity in mice is associated with a shift in the T_h2_/T_h1_ balance that leads to eosinophil-mediated multi-organ inflammation (Ji et al., 2007).

The successful use of PI3Kγ/δ inhibitors to treat inflammatory autoimmune diseases must take into account the need to balance suppression of autoimmune responses with maintenance of protective immunity as well as the potential for unexpected pro-inflammatory consequences of PI3Kγ/δ inhibition, as has been observed in mice. As mentioned above, eosinophil-mediated inflammation is associated with the loss of both PI3Kγ and PI3Kδ activity in mice (Ji et al., 2007). Moreover, loss of PI3Kδ activity in mice greatly enhances isotype switching to IgE (Zhang et al., 2008, 2012), which could lead to atopic reactions. It remains to be seen whether these pro-inflammatory consequences of PI3Kγ/δ inhibition occur in humans. The paradoxical effects of PI3Kδ inhibition on Tregs, cells that play a critical role in limiting autoimmune reactions, may also be an important factor to consider. Inhibition of PI3Kδ activity has been reported to impair Treg function, even though it promotes Treg development (Patton et al., 2006; Sauer et al., 2008; Okkenhaug and Fruman, 2010). Despite these caveats, phase I clinical trial data have shown that the PI3Kδ inhibitor GS-1101 is safe and well-tolerated by patients (Kahl et al., 2010). Thus optimized dosing regimens that cause partial inhibition of PI3Kγ/δ may have the ability to substantially inhibit autoimmune reactions without rendering protective immune responses ineffective or causing significant unwanted side effects.

## PI3Kδ as a target for treating B-cell malignancies

In addition to a key role for PI3Kδ in autoimmune inflammation, this pathway has emerged as a central mechanism underlying the survival and expansion of various malignant B-cells. BCR signaling is a central pathologic mechanism in B-cell malignancies, including chronic lymphocytic leukemia (CLL), diffuse large B-cell lymphoma (DLBCL) (Davis et al., 2010), and mantle cell lymphoma (MCL). Comparative gene expression profiling data demonstrate that BCR signaling is the most prominent pathway activated in CLL cells isolated from lymphatic tissues (Herishanu et al., 2011). Similarly, MCL cells display signs of constitutive activation of BCR (Rinaldi et al., 2006; Cecconi et al., 2008; Pighi et al., 2011) and PI3K signaling (Martinez et al., 2003; Rizzatti et al., 2005) in the absence of activating mutations. The role of BCR-induced PI3K signaling in promoting the survival of malignant B cells, combined with the fact that PI3Kδ is the predominant PI3K isoform involved in BCR signaling, make this PI3K isoform a very promising target for the treatment of B-cell cancers. Substantial progress, both preclinical and clinical, has been made with GS-1101 (CAL-101), a selective inhibitor of the PI3Kδ pathway. The importance of the PI3Kδ pathway in B cells, as well as the initial development of GS-1101 as a treatment for B-cell malignancies, has been reviewed recently by Fruman and Rommel (2011).

### Role of PI3Kδ signaling in B-cell malignancies

The potential role of excessive PI3K signaling in the development of B-cell malignancies was initially reported by Borlado et al. (2000), who showed that mice expressing a constitutively active form of PI3K develop infiltrating lymphoproliferative disorders as well as autoimmune disease. There is now mounting evidence for the importance of PI3K signaling in B-cell malignancies. Uddin et al. (2006) demonstrated the constitutive activation of the PI3K pathway in several DLBCL cell lines, importantly, in primary cells from the majority of patients with DLBCL. Constitutive activation of the PI3K pathway, as measured by elevated levels of phospho-Akt, has also been demonstrated in MCL cell lines and in primary cells from patients with MCL (Rudelius et al., 2006). PI3K activation has also been observed in follicular lymphoma (FL), mediastinal DLBCL, and Hodgkin's lymphoma (Renne et al., 2007; Garcia-Martinez et al., 2011). More recently, a role for the PI3Kδ isoform in the pathophysiology of B-cell malignancies has emerged. Several experiments have shown that there is excessive p110δ activity in malignant lymphoid cells. Herman et al. (2010) showed that there were significantly higher levels of PI3K p110δ activity in primary cells from patients with CLL than in normal hematopoietic cells. Similarly, PI3K p110δ is hyperactivated in plasma cell myeloma (PCM) cell lines and malignant cells from patients with PCM (Ikeda et al., 2010). Excessive PI3Kδ activity has also been observed in cell lines and primary cells from patients with Hodgkin's lymphoma (Meadows et al., 2012).

The development of GS-1101, a potent and selective inhibitor of PI3K p110δ, has enhanced our understanding of the role of p110δ in B-cell malignancies. GS-1101 (5-Fluoro-3-phenyl-2-[(S)-1-(9H-purin-6-ylamino)-propyl]-3H-quinazolin-4-one) inhibits PI3K p110δ with an IC_50_ value of 2.5 nM *in vitro* whereas its IC_50_ values for the p110α, p110β and p110γ subunits are 820, 565 and 89 nM, respectively (Lannutti et al., 2011). GS-1101 also shows a greater selectivity for inhibition of p110δ than for mTOR and other related kinases, and demonstrates high selectivity for p110δ when tested against a panel of more than 400 diverse kinases (Lannutti et al., 2011). Additionally, in isoform-specific cell-based assays, GS-1101 blocks PI3Kδ-dependent responses at concentrations that are >240-fold lower than those required to inhibit responses that are dependent on other PI3K isoforms (Lannutti et al., 2011). In addition to GS-1101, several other PI3Kδ inhibitors are now in clinical trials and many more are in preclinical development, as described in a recent review (Norman, 2011).

The mechanism of action of GS-1101 has been studied in cell lines and primary patient samples representing diverse B-cell malignancies including CLL, DLBCL, and multiple myeloma (MM) (Herman et al., 2010; Ikeda et al., 2010; Hoellenriegel et al., 2011; Lannutti et al., 2011). Primary cells derived from CLL patients have high levels of p110δ and treating these cells with low micromolar concentrations of GS-1101 promotes apoptosis through caspase activation (Herman et al., 2010). This cytotoxic effect of GS-1101 is independent of the IgV_H_ mutational status of the cells as well as interphase cytogenetic abnormalities, the common prognostic factors associated with poor response to therapy in CLL. Interestingly, GS-1101 inhibits not only constitutive PI3K signaling in these cells but also antagonizes CLL cell survival by blocking the protective effect of CD40-ligand (CD40L) and microenvironmental stimuli (Herman et al., 2010). In particular, the cytokines BAFF and TNFα enhance the survival of primary CLL cells *in vitro* but this effect is attenuated by submicromolar concentrations of GS-1101 (Herman et al., 2010). Similarly, the survival advantage conferred to CLL cells by growth on fibronectin or stromal cell layers is reversed by GS-1101 (Herman et al., 2010).

More recent work demonstrated that GS-1101 completely blocks survival signals mediated by BCR engagement and significantly reduces survival of CLL cells cultured on specialized nurse-like cells (NLC) derived from peripheral blood monocytes (Hoellenriegel et al., 2011). Furthermore, GS-1101 decreases the secretion of survival-associated chemokines by both the CLL cells (CCL2, CCL3) and stromal cells (CXCL13) in a co-culture model. The co-culture of CLL and NLC triggers the release of additional survival factors (CCL7, CCL17, CCL22, soluble CD40L, IL-6, and TNFα) whose secretion is also reduced by GS-1101. Additionally, GS-1101 inhibits CLL cell chemotaxis toward CXCL12 and CXCL13. These chemokines are commonly present in the B-cell follicles of lymph nodes and chemoattract CLL cells to this protective microenvironment. Together these observations suggest that PI3Kδ inhibition with GS-1101 can antagonize intrinsic survival signals and also block survival signals provided by extrinsic factors in the tumor microenvironment.

PI3Kδ plays an essential role in PCM cell function (Ikeda et al., 2010). All PCM cell lines express p110δ and treatment with micromolar concentrations of GS-1101 inhibits the constitutive phosphorylation of Akt observed in these cells. This correlates with caspase-dependent apoptosis in p110δ-positive PCM cells, with minimal cytotoxicity in p110δ-negative cells (Ikeda et al., 2010). IL-6 and IGF-1, which are present in the bone marrow (BM) microenvironment, promote MM cell proliferation and survival. Importantly, the cytotoxic effect of GS-1101 on PCM cells was not diminished in the presence of IL-6 and IGF-1, suggesting that GS-1101 can overcome the protective effects of these cytokines within the BM milieu. GS-1101 also exhibits an inhibitory effect on PCM cells in the presence of BM stromal cells (BMSCs), and inhibited the growth, cytokine production, and Akt phosphorylation that are induced in PCM cells by their association with BMSCs (Ikeda et al., 2010).

### PI3K p110δ inhibition with GS-1101 in patients with B-cell malignancies

GS-1101 was the first selective inhibitor of PI3Kδ to enter clinical testing for various B-cell malignancies including CLL, non-Hodgkin lymphoma (NHL), acute myeloid leukemia (AML), DLBCL, and MM. Remarkably, treatment with GS-1101 as a single agent provided durable remissions to a significant percentage of patients with CLL and certain subtypes of NHL (Sharman et al., 2011). Most of these patients had relapsed from multiple other treatment regimens yet responded to the p110δ inhibitor. GS-1101 also showed impressive efficacy when combined with standard-of-care agents (e.g., rituximab, bendamustine, and fludarabine) for indolent NHL (iNHL) and CLL. Currently GS-1101 has advanced to phase 3 clinical testing in CLL as a single agent or in combination with rituximab and bendamustine (clinical trial identifiers: NCT01569295, NCT01539291, and NCT01539512). In addition, an ongoing phase 2 clinical study is evaluating the efficacy and safety of GS-1101 in patients with relapsed or refractory Hodgkin lymphoma (clinical trial identifier: NCT01393106).

Although most of the data have not yet been published in peer-reviewed journals, some of the clinical data for trials involving GS-1101 have been presented at meetings over the past few years and described in abstracts of the conference proceedings (Flinn et al., 2009a,b,c; Brown et al., 2010; Flinn et al., 2010; Furman et al., 2010a; Kahl et al., 2010; de Vos et al., 2011; Kahl et al., 2011; Leonard et al., 2011; Sharman et al., 2011). Most recently, Miller and colleagues (Sharman et al., 2011) reported on a phase I study of GS-1101 as single agent and in combination with rituximab and/or bendamustine in patients with relapsed/refractory CLL (clinical trial identifiers: NCT00710528 and NCT01088048). Data were presented from 55 patients with CLL who were administered GS-1101 monotherapy and 54 patients who received GS-1101-based combination therapies. GS-1101 as single agent or as part of a combination therapy caused lymph node shrinkage in a large majority of CLL patients (>79%) across all dose levels. Nodal size changes occurred in two phases: a steep initial reduction (>50% reduction by week 8) that was followed by a persistent continuing decline over several months. The extent and kinetics of the nodal responses were similar in patients treated with single-agent GS-1101 or with combination therapies. A significant percentage of patients showed durable responses (approximately 25% overall response rate) with single-agent GS-1101 and remained on study for many cycles of treatment. GS-1101-based combinations substantially increased the overall response rate to 81% compared to single-agent therapy. The median progression-free survival in patients on GS-1101 monotherapy was >12 months and the median progression-free survival for any combination therapy has not yet been reached but has exceeded 12 months.

The initial lymph node reduction observed in the majority of CLL patients treated with single-agent GS-1101 was accompanied by a transient elevation in circulating lymphocyte counts (Sharman et al., 2011), a phenomenon known as lymphocytosis. This lymphocytosis resolved over several months of continued GS-1101 treatment. This redistribution of CLL cells from the tissues into the blood is likely the result of lymphocytes being released from lymphoid tissue microenvironments or failing to home from the blood into lymph nodes, resulting in the eventual death of tumor cells that are prevented by GS-1101 from accessing the supportive microenvironment of the lymph nodes. Importantly, the pattern and extent of lymphocytosis in CLL patients were altered by combination therapy. The combination of GS-1101 with rituximab or fludarabine led to a shorter duration of lymphocytosis while the combination with bendamustine largely eliminated the increase in circulating lymphocyte counts (Sharman et al., 2011).

The CLL patients treated with GS-1101 monotherapy and combination therapy also showed significantly reduced plasma levels of the chemokines CXCL13, CCL3, CCL4 and cytokine TNFα after 28 days of treatment compared with pretreatment measurements, further suggesting disruption of the CLL microenvironment by GS-1101 (Hoellenriegel et al., 2011; Sharman et al., 2011). As described earlier, previous studies demonstrated an important role for PI3Kδ in B-cell adhesion, migration, and homing to lymphatic tissues (Reif et al., 2004; Durand et al., 2009). B cells from PI3K p110δ knockout mice respond poorly to CXCL13, and exhibit reduced homing to lymphatic tissues in adoptive transfer experiments (Reif et al., 2004). PI3Kδ activity is also required for chemotactic and adhesive signals that mediate the localization of B cells in lymphoid tissue, as indicated by the ability of *in vitro* IC87114 treatment to inhibit B cell chemotaxis toward CXCL13 and S1P as well as CXCL13-stimulated adhesion to ICAM-1, which is mediated by the LFA-1-integrin (Durand et al., 2009). Consistent with this, treating mice with IC87114, a selective PI3Kδ inhibitor (Sadhu et al., 2003b), disrupted the *in vivo* localization of MZ B cells in mice (Durand et al., 2009). Indeed, the notable clinical activity of GS-1101 in CLL is associated with mobilization of CLL cells from tissues into the blood, which results in pronounced lymph node shrinkage and transient lymphocytosis. Thus GS-1101 displays a dual mechanism of action against CLL, directly decreasing BCR-induced CLL cell survival (see above) and inhibiting chemokine-dependent interactions that retain CLL cells in survival-promoting tissue microenvironments. These combined actions may account for the ability of this PI3Kδ inhibitor to sensitize CLL cells to the current standard-of-care agents. Similar effects have been observed with inhibitors of the Syk, Btk, and mTOR kinases, which also function in both chemokine receptor and BCR signaling pathways (Friedberg et al., 2010; Zent et al., 2010; Burger, 2012; de Rooij et al., 2012). The common mechanism underlying the antitumor activity of these kinase inhibitors that are active against CLL may be their ability to inhibit multifunctional signaling pathways that control leukemia cell survival, adhesion, migration, and homing.

Similar promising results have been obtained in a phase 1 study (de Vos et al., 2011) of GS-1101 as a single agent and in combination with rituximab and/or bendamustine for treatment of patients with relapsing/remitting iNHL (clinical trial identifiers: NCT00710528 and NCT01088048). Data were presented from 63 patients with iNHL on GS-1101 monotherapy and 52 patients on GS-1101-based combination therapies representing all four subtypes of iNHL: follicular, small lymphocytic, lymphoplasmacytic, and MZ, with FL being the most common in all treatment arms. GS-1101 as single agent or as combination therapy caused substantial tumor regression in a large majority of patients with indolent NHL and across all dose levels. The overall response rate for GS-1101 monotherapy was 38% across all dose levels and 59% at doses ≥ 100 mg twice daily. GS-1101-based combinations substantially increased the overall response rate to 83% compared to single-agent therapy. Furthermore, complete remission was achieved in 15–25% of patients on GS-1101-based combination therapies. Similar to clinical data in CLL, both GS-1101 monotherapy and combination therapy were associated with durable tumor control with the median progression-free survival in patients receiving GS-1101 monotherapy at doses ≥ 100 mg twice daily, receiving any combination therapy, was >12 months.

Consistent with observations in CLL patients, treatment of iNHL patients with single-agent GS-1101 or combinations significantly decreased plasma levels of CCL17, CCL22, CXCL13, and TNFα. However, unlike the biphasic nodal response and transient lymphocytosis observed in CLL patients treated with GS-1101 monotherapy, iNHL patients show profound and rapid reductions in lymph nodes. The lymphocytosis has not been reported in patients with iNHL treated with single-agent GS-1101 or with combination therapies, suggesting that redistribution of cells to the periphery may not be a critical mechanism of antitumor activity of GS-1101 in iNHL. This is also consistent with the observation that iNHL cells are not generally found in circulation; hence, they are likely being cleared in the tissue sites rather than in the periphery.

GS-1101 has also been tested as a single agent treatment in a small number of patients (9–11 each) with MM, AML, and DLBCL malignancies. However, as reported in conference proceeding abstracts, it has not shown the same level of efficacy as for CLL and NHL (Flinn et al., 2009a; Furman et al., 2010b; Kahl et al., 2010). Because the effects of GS-1101 on the redistribution of malignant cells and on plasma chemokine and cytokine levels in these patients has not been reported, it is not known if the lack of efficacy was due to insufficient inhibition of the PI3K pathway or to protective microenvironmental signals in these malignancies. Whether GS-1101 treatment would make these malignant cells more susceptible to other standard-of-care treatments remains to be determined.

An important consideration for therapeutics that target signaling pathways that are essential for the survival and dissemination of malignant lymphocytes is whether they will also suppress beneficial anti-tumor immunity. Several studies have reported that PI3Kδ is required for the function of immune cells that could influence the immune response to tumor cells. GS-1101 strongly suppresses cytokine production by human T cells (Herman et al., 2010). Moreover, inhibition of PI3Kδ may also have detrimental effects on tumor immunosurveillance by NK cells and cytotoxic T lymphocytes (CTL). PI3Kδ activity is important for NK cell activation and function (Kim et al., 2007; Saudemont et al., 2007) and for induction of both the perforin-granzyme and death-receptor pathways of CTL-mediated tumor cell killing (Putz et al., 2012). In addition, Tregs play a key role in restraining immune responses including anti-tumor responses but the net effect of PI3Kδ inhibition on Tregs is still unclear. Loss of PI3Kδ activity promotes the differentiation of T cells into Tregs but can also inhibit Treg function (Patton et al., 2006; Sauer et al., 2008; Okkenhaug and Fruman, 2010). Thus determining how to balance inhibition of malignant cell survival with maintenance of anti-tumor immunity will be the key to treatment success.

## Conclusions

Recent data has demonstrated that the PI3Kδ pathway is a critical signaling circuit in B cells and plays a key role in autoimmune inflammation as well as some lymphoid malignancies. This pathway is hyperactivated in various B-cell malignancies and its inhibition with PI3Kδ-targeted compound has achieved promising clinical responses in patients with CLL, iNHL, and other B-cell neoplasia. The PI3Kδ inhibitor GS-1101 appears to antagonize both intrinsic and extrinsic cell survival signals, decreases the survival of CLL cells directly, and abrogates cellular interactions between CLL cells and components of the tissue microenvironment that normally sustain leukemia and lymphoma cells in a protective niche. Ongoing clinical studies are further characterizing the risks and benefits of long-term PI3Kδ inhibition. Nevertheless, the initial trials suggest that GS-1101 has significant potential both as a single agent and in combination with bendamustine and rituximab for the treatment of CLL and iNHL (clinical trial identifiers: NCT01569295, NCT01539512, and NCT01539291). Ongoing clinical studies are also evaluating GS-1101 to potentially expand its utility in additional B-cell malignancies such as Hodgkin's lymphoma (clinical trial identifier: NCT01393106).

The PI3Kδ pathway is also constitutively activated in various inflammatory conditions and pharmacological inhibition or genetic inactivation of this pathway has demonstrated efficacy in several preclinical models of inflammation. Clinical studies are now evaluating the potential of PI3Kδ inhibitors for the treatment of a variety of inflammatory diseases (clinical trial identifiers: NCT00836914 and NCT01462617). In addition to B-cell inhibition, PI3Kδ inhibitors are also likely to significantly impact differentiation, migration, and cytokine production by T cells as well as the degranulation of mast cells, suggesting that these agents may be effective beyond autoimmune diseases. As is the case for all immunosuppressive agents, a balance between suppressing autoimmunity and maintaining protective immunity needs to be established. Orally available small molecule inhibitors that have a short *in vivo* half-life would allow this balance to be adjusted in a continual manner.

In conclusion, PI3Kδ plays a major role in both B-cell-mediated inflammation and B-cell malignancies, and PI3Kδ inhibitors therefore represent a promising therapeutic approach to treating these diseases.

### Conflict of interest statement

Kamal D. Puri is an employee of Gilead Sciences Inc., the manufacturer of IC87114 and GS-1101. Michael R. Gold has no commercial or financial relationships that could be construed as a potential conflict of interest.
